# Characterization of Bacterial Communities of Cold-Smoked Salmon during Storage

**DOI:** 10.3390/foods10020362

**Published:** 2021-02-07

**Authors:** Aurélien Maillet, Pauline Denojean, Agnès Bouju-Albert, Erwann Scaon, Sébastien Leuillet, Xavier Dousset, Emmanuel Jaffrès, Jérôme Combrisson, Hervé Prévost

**Affiliations:** 1 Biofortis Mérieux NutriSciences, 3 route de la Chatterie, 44800 Saint-Herblain, France; erwann.scaon@mxns.com (E.S.); sebastien.leuillet@mxns.com (S.L.); jerome.combrisson@free.fr (J.C.); 2 INRAE, UMR Secalim, Oniris, Route de Gachet, CS 44307 Nantes, France; pauline.denojean@mxns.com (P.D.); agnes.bouju@oniris-nantes.fr (A.B.-A.); xavierdousset@yahoo.fr (X.D.); emmanuel.jaffres@oniris-nantes.fr (E.J.); herve.prevost@oniris-nantes.fr (H.P.)

**Keywords:** seafood products, cold-smoked salmon, processing plant, bacteria, metabarcoding, microbiota, spoilage

## Abstract

Cold-smoked salmon is a widely consumed ready-to-eat seafood product that is a fragile commodity with a long shelf-life. The microbial ecology of cold-smoked salmon during its shelf-life is well known. However, to our knowledge, no study on the microbial ecology of cold-smoked salmon using next-generation sequencing has yet been undertaken. In this study, cold-smoked salmon microbiotas were investigated using a polyphasic approach composed of cultivable methods, V3—V4 16S rRNA gene metabarcoding and chemical analyses. Forty-five cold-smoked salmon products processed in three different factories were analyzed. The metabarcoding approach highlighted 12 dominant genera previously reported as fish spoilers: Firmicutes *Staphylococcus, Carnobacterium, Lactobacillus,* β-Proteobacteria *Photobacterium, Vibrio, Aliivibrio, Salinivibrio, Enterobacteriaceae Serratia,* *Pantoea*, γ-Proteobacteria *Psychrobacter, Shewanella* and *Pseudomonas*. Specific operational taxonomic units were identified during the 28-day storage study period. Operational taxonomic units specific to the processing environment were also identified. Although the 45 cold-smoked salmon products shared a core microbiota, a processing plant signature was found. This suggest that the bacterial communities of cold-smoked salmon products are impacted by the processing environment, and this environment could have a negative effect on product quality. The use of a polyphasic approach for seafood products and food processing environments could provide better insights into residential bacteria dynamics and their impact on food safety and quality.

## 1. Introduction

With 175,000 tons produced in the European Union in 2019, cold-smoked salmon (CSS) is a leading fish product with an important trade value (€2.77 billion) [1,2]. CSS is a lightly preserved product with no thermic treatment and is mainly consumed as a ready-to-eat (RTE) food. Due to a large number of intrinsic and extrinsic factors, such as pH, water activity (a_w_), temperature, environmental origins and processing practices, such commodities are highly fragile [3,4]. Salting and smoking are mandatory steps in CSS processing to decrease foodborne pathogens and spoilage risks [5]. As described by Leroi et al. (2000), the purpose of salting and smoking is to decrease the a_w_ through dehydration [6]. The chloride ions from salt additives are also toxic for some microorganisms, and the phenolic compounds produced during the smoking step have a bacteriostatic effect. Smoking is furthermore used to bring out specific tastes and aromas [7].

The CSS bacterial community has been widely studied in the scientific literature aiming to describe spoilage and pathogenic microbiota [5]. Gram-negative bacteria such as *Shewanella putrefaciens, Aeromonas* spp. and marine *Vibrionaceae Photobacterium phosphoreum* have been described as dominating CSS microbiota in the early stages of storage [7].

Gram-positive lactic acid bacteria (LAB: *Lactobacillus, Carnobacterium maltaromaticum*) seem to dominate CSS microbiota at the end of the product’s shelf-life. Paludan-Müller et al. (1998) reported a high number of LAB (10^7^–10^8^ CFU/g). Gram-negative psychrotrophic bacteria *Enterobacteriaceae Serratia liquefaciens* were also reported in some cases to co-dominate the microbiota at the end of the shelf-life [5,8,9]. In addition, *Brochothrix thermosphacta* has already been described as dominating CSS microbiota [10]. As an RTE food product, CSS are often faced with the foodborne pathogenic bacteria *Listeria monocytogenes* [5].

The majority of the studies mentioned were based on culturable approaches. Traditional methods can be time-consuming and lead to technical biases (viable but non-culturable cells, non-specific media and culture conditions) [11]. Due to the challenging storage conditions of a product like CSS (temperature, phenolic compounds due to the smoking step, salt), culturable approaches might be insufficient for studying the entire CSS bacterial community. Culture-independent methods such as fingerprinting (Denaturing Gradient Gel Electrophoresis, Temperature Gradient Gel Electrophoresis) are DNA-based methods which offer tools to monitor the bacterial community on food products and food-associated microbial ecosystems [12,13,14]. More recently, next-generation sequencing (NGS) has offered new ways to explore food microbial ecology [15]. Bacterial diversity can now be assessed through high throughput sequencing approaches which facilitate the identification of microbes and the relative abundance of taxa for a high number of samples in a single analysis [16].

A few studies have sought to assess the CSS bacterial community using DNA-based methods [11,17,18]. Although NGS was previously used to determine contamination of fresh salmon filets, to our knowledge no study of the evolution of the CSS microbial ecology during shelf-life has used this type of approach [19,20]. Yet, NGS could provide an increasingly deeper insight into the microbial diversity of seafood and seafood products [21].

This study used 16S rRNA gene metabarcoding to assess the evolution of bacteria on 45 CSS products from three different factories that were stored for 28 days at two different temperatures (4 °C first week, 8 °C remaining weeks).

A polyphasic approach was implemented in this study; culture-dependent and independent methods associated with chemical analyses were used.

## 2. Materials and Methods

### 2.1. Cold-Smoked Salmon Sampling

Forty-five vacuum-packed CSS, originating from nine different batches and three different French processing factories (referred to henceforth as A, B and C) with a similar use-by date, were collected from local supermarkets. The CSS packs were stored for seven days at 4 °C then 21 days at 8 °C as described by Chaillou et al. (2015), in accordance with the French food aging test standard AFNOR NF V01-003 [22,23]. Details on the samples are summarized in Table 1.

### 2.2. Bacterial Enumeration

From each sample, a 10-g portion of CSS was added to 90 mL of sterile buffered peptone water (25.5 g/L) (Biokar Diagnostics, Allonne, France) to obtain a 10-fold dilution. Samples were homogenized for 2 min in a sterile stomacher plastic bag provided with a 63 μm porosity filter (Interscience, Saint-Nom-la-Bretèche, France) using a stomacher 400 device (Intersciences, Saint-Nom-la-Bretèche, France).

Total psychrotrophic viable counts (TPVC) were enumerated on plate count agar (PCA) medium (Oxoid, Thermo Fisher Diagnostics, Dardilly, France) supplemented with 2% NaCl. The PCA plates were incubated at 15 °C for five to seven days. Lactic acid bacteria (LAB) were enumerated on de Man, Rogosa and Sharpe (MRS) agar plates (bioMérieux, Crapone, France) incubated for two days at 30 °C. *Brochothrix thermosphacta* were investigated on streptomycin sulfate thallous acetate agar (STAA) (Oxoid, Thermo Fisher Diagnostics, Dardilly, France) incubated for two days at 25 °C [24]. *Enterobacteriaceae* were enumerated after two days at 30 °C on violet red bile glucose agar (VRBG) (Biokar Diagnostics, Allonne, France) and marine *Vibrio* were enumerated on marine agar (five days at 25 °C) (Becton Dickinson, Rungis, France). To enumerate bacterial colonies, 100 μL of appropriate dilution in buffered peptone water were spread over the agar. Results were expressed in colony forming unit per gram CSS (CFU/g). Detections limits were 1 and 2 Log CFU/g, respectively, for *Enterobacteriaceae* and other counts.

### 2.3. Chemical Analyses

Total fat, dry matter content, salt content and total phenol were measured as described by Leroi et al. (2015) [25]. Total volatile basic nitrogen (TVBN) and trimethylamine (TMA) were determined in duplicate from 100 g of CSS using the Conway micro-diffusion method [26].

### 2.4. DNA Extraction

DNA were extracted using Qiagen DNeasy PowerFood Microbial (Qiagen, Courtaboeuf, France). A first step of mechanical cell lysis was performed using the glass beads provided and a FastPrep (MPbiomedicals, Illkirch, France) for 30 s at a frequency of 6 m/s. DNA were extracted from three technical replicates from each sample. A Qubit^®^ 2.0 fluorometer using a Qubit^®^ dsDNA BR Assay Kit (Life technologies, Thermo Fisher Scientific, Villebon-sur-Yvette, France) was used to quantify DNA. Additional blank negative controls with no samples were used to exclude DNA contamination during extraction.

### 2.5. 16S rRNA Gene Sequencing

#### 2.5.1. Library Preparation and Sequencing Using Illumina^®^ MiSeq Platform

Briefly, the extracted DNA were PCR amplified to construct a sequencing library targeting the V3—V4 region of the bacterial 16S rRNA gene. PCR reactions were performed using 5 µL of DNA template, 12.5 µL of 2 × Kapa HiFi Hotstart ready mix (Roche, Boulogne-Billancourt, France) and 5 µL of 1 µM primers 341F (5′-CCTACGGGNGGCWGCAG-3′) and 785R (5′-GACTACHVGGGTATCTAATCC-3′) [27]. Amplicons were purified using an Agencourt AMPure kit (Beckman Coulter, Villepinte, France). PCR product concentration and size were checked on a 2100 Bioanalyzer platform using the DNA 7500 kit (Agilent Technologies, Les Ulis, France) and indexed using a Nextera XT DNA Library Prep kit (Illumina, Paris, France) following Illumina recommendations. Samples were then pooled in an equimolar concentration (4 nM) and sequenced through the Illumina^®^ MiSeq platform using a 2 × 250 V2 chemistry kit (Illumina, Paris, France) according to the Illumina^®^ standard operating procedures.

#### 2.5.2. Sequencing Data Processing and Analyses

The count table and taxonomy of the operational taxonomic units (OTUs) were obtained using the FROGS bioinformatic pipeline [28]. Paired-end raw reads were merged using FLASh 1.2.11 with a maximum of 10% mismatch in the overlapped region [29]. Primers were removed using Cutadapt 1.18. Clustering of reads into OTUs (97% identity) was performed using Swarm 2.2.2 [30], and a denoising step was performed. Chimera were then detected and removed using VSearch 1.3.0 [31]. OTUs with less than 5/100,000 of the total number of sequences from the whole dataset were removed [32]. Taxonomy assignments were performed using RDP classifier 2.11 and the Silva 16S rRNA gene database (SSURef_128_SILVA) [33,34,35]. OTUs with a genus affiliation bootstrap threshold < 0.8 were removed.

### 2.6. Statistical Analyses

Statistical analyses and plots were performed in the R environment (v. 3.6.2) [36]. For metabarcoding data, alpha and beta diversity analyses were conducted and relative abundances were determined using the Phyloseq package (1.30.0) and its dependencies [37]. Samples read libraries were rarefied to an even depth (10,000 reads per sample) to be normalized. Permutational multivariate analysis of variance (PERMANOVA) based on a weighted UniFrac distance matrix was carried out using 9999 permutations to detect significant effects/differences in the bacterial community analyzed [38]. UpSet plots were used to assess OTU intersections according to the processing factories and storage date [39]. These plots were generated using the UpSetR package (1.4.0) [40].

The chemical parameters, the relative abundance of each taxon at the genus level, and the alpha diversity metrics were studied using linear mixed models considering the factory, the storage time and their interaction as fixed effects, and the production batch as a random effect. For all endpoints, the *p*-values were adjusted using Tukey’s method for pairwise comparisons between factories at each time point and between time points for each factory. A *p*-value < 0.05 was considered statistically significant.

## 3. Results

### 3.1. Microbiological Analyses

**Figure 1:**
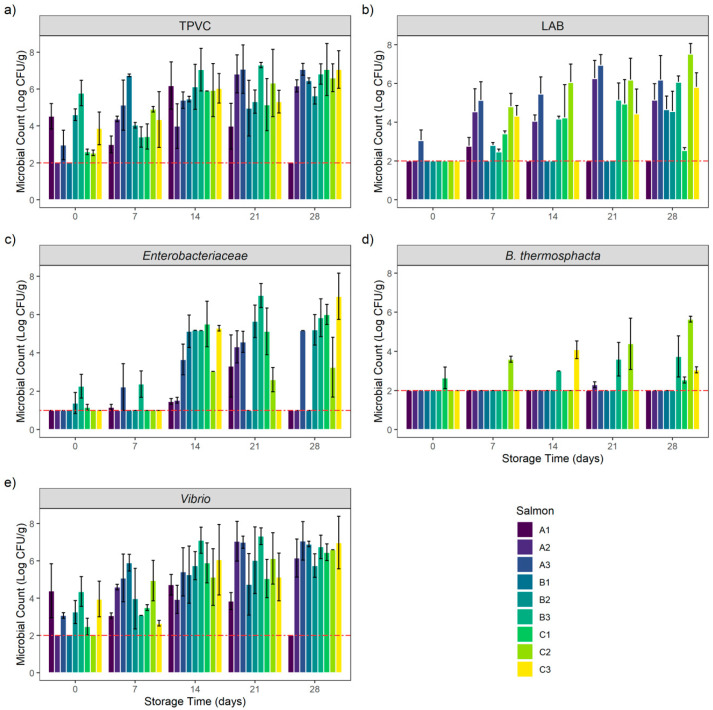
Bacterial growth evolution of (**a**) total psychrotrophic viable count (TPVC), (**b**) lactic acid bacteria (LAB), (**c**) *Enterobacteriaceae*, (**d**) *Brochothrix thermosphacta* and (**e**) *Vibrio* in vacuum-packed cold-smoked salmon (CSS) products during 28 days of storage. Results are expressed in Mean ± SD Log CFU/g of CSS products. The red-dashed line represents the limits of detection: 1 and 2 Log CFU/g, respectively, for *Enterobacteriaceae* and other counts.

Bacterial growth of the nine CSS batches during the 28-day storage period are presented in Figure 1 and summarized in Table S1.

At the beginning of the bacterial kinetic, total psychrotrophic viable counts (TPVC) were heterogenous among the different samples. Except for products A1, B2 and B3, TPVC increased during the storage period to reach D28 counts between 5.64 ± 0.45 and 7.07 ± 0.32 Log CFU/g. Interestingly, TPVC on products A1, B2 and B3 were high at the beginning of the experiment (D0) (between 4.53 ± 0.69 and 5.78 ± 0.69 Log CFU/g) and remained stable during the storage period. The A1 sample count at D28 was below the enumeration limit (<2 Log CFU/g).

Lactic acid bacteria (LAB) counts were low at the beginning of the experiment (D0). Except for product A3, which had an enumeration of 3.06 ± 0.55 Log CFU/g, all counts were below the enumeration limit. This microbial group quickly grew and reached its maximum after 21 days of storage. Interestingly, product A1’s count was low or below the enumeration limit during the entire storage period with a maximum at D7 (2.77 ± 0.45 Log CFU/g). We observed the same situation on B1 and B2 products. However, these two samples reached respectively 4.66 ± 0.69 and 4.55 ± 1.05 Log CFU/g after 28 days of storage.

*Enterobacteriaceae* initial enumerations (D0) were low or below the enumeration limit (<1 Log CFU/g). Between 1.17 ± 0.15 and 2.27 ± 0.62 Log CFU/g were counted on products B2, B3 and C1 at the beginning of the storage period. *Enterobacteriaceae* counts then increased during storage on products A3, B2, B3 and C1 to reach a maximum at D28 (between 5.21 ± 0.8 Log CFU/g for B2 and 6.96 ± 1.21 Log CFU/g for C1). Product C3 counts after 7 and 21 days of storage were below the enumeration limit, whereas 5.29 ± 0.15 Log CFU/g and 6.96 ± 1.21 Log CFU/g were enumerated at D14 and D28. The same situation was observed on product B1: all counts were below the enumeration limit except for D14 with a count of 5.13 ± 0.85 Log CFU/g. In addition, this trend was observed on product A2: all counts were low except for D21 with a count of 4.32 ± 0.84 Log CFU/g. *Enterobacteriaceae* counts on products A1 and C2 increased slowly to reach a maximum of 3.32 ± 1.62 Log CFU/g at D21 for A1 and 3.26 ± 1.56 Log CFU/g at D28 for C2. The A1 sample count at D28 was below the enumeration limit.

For *Brochothrix thermosphacta*, the initial enumerations (D0) were below the enumeration limit on all samples except for product C1, with an enumeration of 2.65 ± 0.55 Log CFU/g. *B. thermosphacta* counts were below the enumeration limit during the entire storage period on products A1, A3, B1 and B2. The same situation also was initially observed on product A2; however, 2.3 ± 0.15 Log CFU/g were enumerated on this product at D21.

*B. thermosphacta* was then counted on products B3 and C2, with an increase during the storage period to reach a maximum of respectively 3.74 ± 1.05 and 5.64 ± 0.15 Log CFU/g at D28. Product C1 counts remained stable during the storage period. The count was below the enumeration limit from D7 to D21 to reach 2.54 ± 0.15 Log CFU/g at D28. The same situation was encountered on product C3. Counts were below the enumeration limit at D0, D7 and D21 but 4.08 ± 0.45 and 3.06 ± 0.15 Log CFU/g were enumerated respectively at D14 and D28.

*Vibrio* initial counts (D0) were high (between 3.07 ± 0.15 Log CFU/g on product A3 and 4.39 ± 1.45 Log CFU/g on product A1) on all products except for A2, B2 and C2, where the counts were below the enumeration limit. Except for product A1, *Vibrio* counts increased during the storage period to reach a maximum count after 21 and 28 days of storage (between 6.03 ± 1.79 Log CFU/g at D21 on product B2 and 7.07 ± 1.03 Log CFU/g at D28 on product A3). Globally, *Vibrio* counts followed the same trend as TPVC. Product A1 *Vibrio* counts were stable during 21 days of storage with counts between 3.06 ± 0.15 and 4.72 ± 0.55 Log CFU/g. The A1 sample count at D28 was below the enumeration limit.

### 3.2. Chemical Analyses

**Figure 2:**
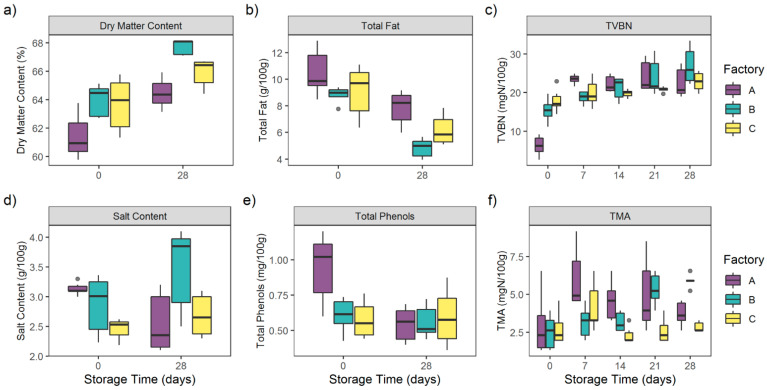
Evolution of (**a**) dry matter content (%), (**b**) total fat (g/100 g), (**c**) total volatile basic nitrogen (TVBN) (mgN/100 g), (**d**) salt content (g/100 g), (**e**) total phenols (mg/100 g), (**f**) trimethylamine (TMA) (mgN/100 g) of 45 CSS products during 28 days of storage.

The evolution of the chemical components of each CSS sample during the 28 days of storage is represented in Figure 2.

Dry matter content among all of the CSS samples significantly increased during storage (*p* < 0.0001) from 63.32 ± 1.86% to 66.25 ± 1.55%. Interestingly, no significant differences in the dry matter content between the three factories’ samples were observed (*p* = 0.07).

Contrary to dry matter, total fat among all CSS samples significantly decreased during storage (*p* < 0.0001) from 9.47 ± 1.65 g/100 g to 6.29 ± 1.62 g/100 g.

No significant differences in total fat content were observed for the three factories’ samples (*p* = 0.08).

Total phenols, issued from the cold-smoking step, were homogeneous among the different factories’ samples (*p* = 0.46). The total phenols rate among the 45 samples decreased from 0.71 ± 0.24 mg/100 g at D0 to 0.57 ± 0.15 mg/100 g at D28. This difference was only due to the significant decrease (*p* < 0.0001) of A samples’ total phenols from 0.94 ± 0.24 mg/100 g at D0 to 0.55 ± 0.12 mg/100 g at D28.

As far as salt content was concerned, no significant differences were observed among the different factories’ samples (*p* = 0.55) or during storage (*p* = 0.18). Indeed, this parameter was stable throughout the storage period, from 2.89 ± 0.41 g/100 g at D0 to 2.91 ± 0.67 g/100 g at D28.

Spoilage markers TVBN and TMA were also measured at each storage date.

TVBN globally increased during the storage period from 13.17 ± 5.81 mgN/100 g at D0 to 24.09 ± 4.19 mgN/100 g at D28 (*p* < 0.0001). TVBN concentrations were also homogeneous among the different factories (*p* = 0.61).

TVBN increased significantly within A samples, from 6.22 ± 2.51 mgN/100 g at D0 to 23.47 ± 1.2 mgN/100 g at D7 (*p* < 0.0001). Concentrations were then homogeneous from D7 to D28 (*p* > 0.05). For B samples, TVBN were stable at D0 and D7 (*p* = 0.68), with respectively 15.39 ± 2.95 mgN/100 g and 18.78 ± 1.58 mgN/100 g. The concentrations then significantly increased to reach a maximum of 26.96 ± 4.78 mgN/100 g at D28 (*p* < 0.05).

TVBN concentrations of C samples were homogeneous during the storage period (*p* > 0.05), from 17.9 ± 2.98 mgN/100 g at D0 to 22.81 ± 2.49 mgN/100 g at D28.

TMA followed the TVBN trend with a significant increase from 2.73 ± 1.39 mgN/100 g at D0 to 4.15 ± 1.42 mgN/100 g at D28 (*p* < 0.0001). Interestingly, TMA concentrations differed significantly among the different factories’ samples (*p* < 0.0001).

TMA increased significantly within A samples, from 2.95 ± 2.02 mgN/100 g at D0 to 6.0 ± 2.0 mgN/100 g at D7 (*p* = 0.011). Concentrations were then homogeneous from D7 to D28 (*p* > 0.05).

For B samples, TMA were stable at D0 and D7 (*p* = 0.68), with respectively 2.51 ± 0.12 mgN/100 g and 3.16 ± 1.04 mgN/100 g. The concentrations then significantly increased to reach a maximum of 5.89 ± 0.41 mgN/100 g at D28 (*p* < 0.05).

The TMA concentrations of C samples were homogeneous during the storage period (*p* > 0.05), from 2.73 ± 1.03 mgN/100 g at D0 to 2.83 ± 0.33 mgN/100 g at D28.

### 3.3. Metabarcoding Analyses

Out of over 135 samples, six DNA samples could not be amplified and sequenced: A1 at D28 and B2 at D28. A total of 3,584,463 reads passed filters applied through the FROGS pipeline workflow with an average of 27,787 reads/sample ± 27,189 reads.

**Figure 3:**
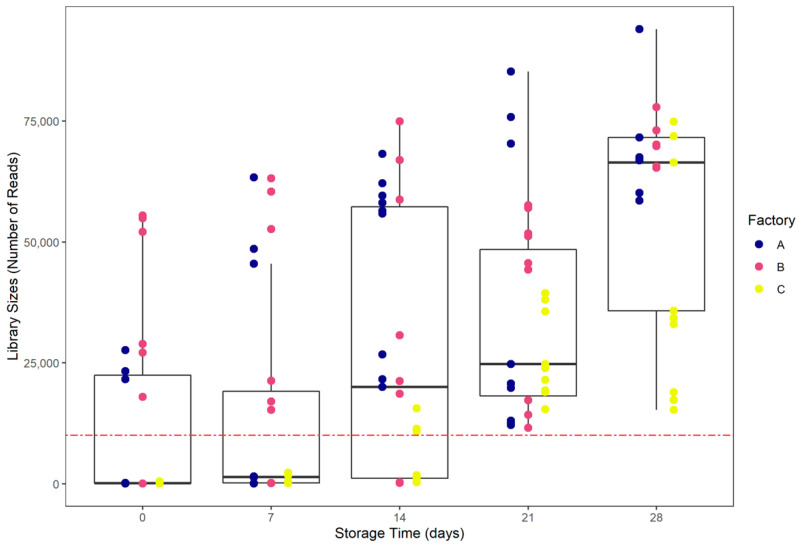
Library sizes distribution according to storage date. The red-dashed line represents a 10,000 reads threshold.

The sizes of the libraries were highly heterogenous. Interestingly, library size increased simultaneously with storage time (Figure 3). Bacterial growth during storage impacted the number of reads (*p* < 0.05). Library sizes were higher at D28 with an average of 57,552 reads ± 22,263. The library sizes of some D0, D7 and D14 samples were too low, and were considered as not being representative of the microbiota of interest. Thus, due to a low number of reads (<10,000) over 15 triplicates (45 samples) were removed for statistical purposes and were not taken into account in any microbial ecology analyses. The other 84 samples were rarefied to an even depth of 10,000 reads and used for microbial ecology analyses.

A total of 56 OTUs were identified and agglomerated in 19 genera including 12 dominants. Dominant populations among all samples were represented by Firmicutes *Staphylococcus* (5.48 ± 10.8%), *Carnobacterium* (18.9 ± 32.3%), *Lactobacillus* (5.24 ± 17.2%), β-Proteobacteria *Photobacterium* (30.4 ± 43.5%), *Vibrio* (6.79 ± 24.6%), *Aliivibrio* (2.55 ± 13.3%), *Salinivibrio* (5.71 ± 20.7%), *Enterobacteriaceae Serratia* (6.8 ± 18.8%), *Pantoea* (3.6 ± 11.7%), γ-Proteobacteria *Psychrobacter* (6.43 ± 18.2%), *Shewanella* (4.75 ± 17.5%) and *Pseudomonas* (2.92 ± 10.4%).

**Figure 4:**
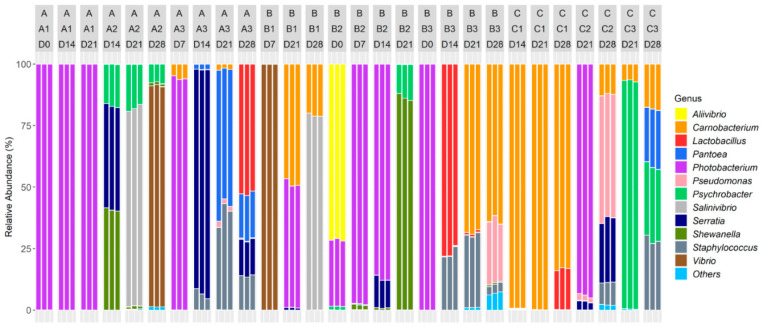
Relative abundance of bacterial genera of vacuum-packed cold-smoked salmon products stored during 28 days (D0, D7, D14, D21, D28). Three different production batches (e.g., A1, A2, A3) were processed in three different processing factories (A, B, C). Only 84 samples are represented: 6 DNA could not be amplified and 45 outliers were removed due to a low number of reads (<10,000). The removed samples were identified in some D0, D7, D14 and D28 samples. Taxa present on average in all samples at a threshold ≥0.5% or having a 90th percentile ≥0.5% are individually represented. In other cases, taxa are grouped and labeled “Others”.

The relative abundances at the genus level are represented in Figure 4.

Genera initially shared a homogeneous repartition among the CSS originating from the three different processing environments (*p* > 0.05). However, the relative abundances significantly differed during the storage period (*p* < 0.001). Indeed, the samples had different dominant populations.

*Photobacterium* and *Aliivibrio* dominated all D0 microbiotas (with respectively 75.66 ± 36.44% and 23.82 ± 35.72%).

After seven days of storage, the bacterial communities were dominated by *Photobacterium* (63.95 ± 47.96%), *Vibrio* (33.35 ± 49.92%) and *Carnobacterium* (1.89 ± 2.87%).

After 14 days of storage, dominant genera were *Photobacterium* (31.27 ± 45.51%) and *Carnobacterium* (16.55 ± 38.05%). Five other genera emerged: *Staphylococcus* (4.94 ± 8.76%), *Lactobacillus* (12.77 ± 29.37%), *Serratia* (24.23 ± 34.40%), *Shewanella* (6.96 ± 15.58%) and *Psychrobacter* (2.81 ± 6.44%).

After 21 days of storage, the microbiotas were dominated by *Photobacterium* (27.20 ± 41.25%), *Psychrobacter* (13.79 ± 29.19%), *Shewanella* (9.73 ± 27.64%) and *Staphylococcus* (7.62 ± 14.73%). Two genera also emerged at D21: *Pantoea* (6.31 ± 18.21%) and *Salinivibrio* (8.95 ± 25.79%).

At the end of the storage period (28 days) *Carnobacterium* (28.32 ± 30.68%), *Lactobacillus* (9.89 ± 18.79%), *Pantoea* (5.99 ± 9.76%), *Pseudomonas* (10.92 ± 18.88%), *Salinivibrio* (11.33 ± 28.38%), *Serratia* (5.77 ± 9.82%), *Vibrio* (12.85 ± 32.23%), *Staphylococcus* (7.88 ± 10.01%) and *Psychrobacter* (5.35 ± 10.61%) dominated the microbiotas.

Except for *Salinivibrio*, the *Vibrionaceae* ratio decreased during storage: *Photobacterium* relative abundance was reduced on A samples between D7 and D28 (*p* < 0.0001). The *Aliivibrio* proportion significantly changed on B samples (*p* < 0.05) and decreased during the storage period (*p* < 0.0001). *Vibrio* relative abundance decreased from D14 to D28 on A products (*p* < 0.0001) and from D7 to D28 on B samples (*p* < 0.05). As far as *Salinivibrio* is concerned, the relative abundance increased from D14 to D28 on A products (*p* < 0.05) and from D7 to D28 on B samples (*p* < 0.0001).

Firmicutes did not share the same evolution: the *Carnobacterium* ratio increased during storage, especially from D0 to D21 on B samples (*p* < 0.0001), whereas the *Lactobacillus* relative abundance increased between D0 and D14 and then decreased from D14 to D28 on B samples (*p* < 0.0001). Moreover, the *Lactobacillus* proportion increased from D7 to D28 on A products (*p* < 0.05). In addition, the *Staphylococcus* ratio increased between D7 to D21 on A samples (*p* < 0.0001) and from D21 to D28 on C salmons.

As far as the *Enterobacteriaceae* family is concerned, the *Serratia* relative abundance increased from D0 to D14 on A CSS (*p* < 0.05) and then was reduced during the remaining period (*p* < 0.0001). The *Pantoea* ratio increased between D7 and D28 on A products (*p* < 0.05).

Globally, γ-Proteobacteria increased during the storage: the *Shewanella* proportion increased between D0 and D21 on B products (*p* < 0.001). The *Psychrobacter* ratio increased significantly from D21 to D28 on C salmons (*p* < 0.0001). The *Pseudomonas* relative abundance increased between D21 and D28 C samples (*p* < 0.0001) and from D0 to D28 on B products (*p* < 0.05).

**Figure 5:**
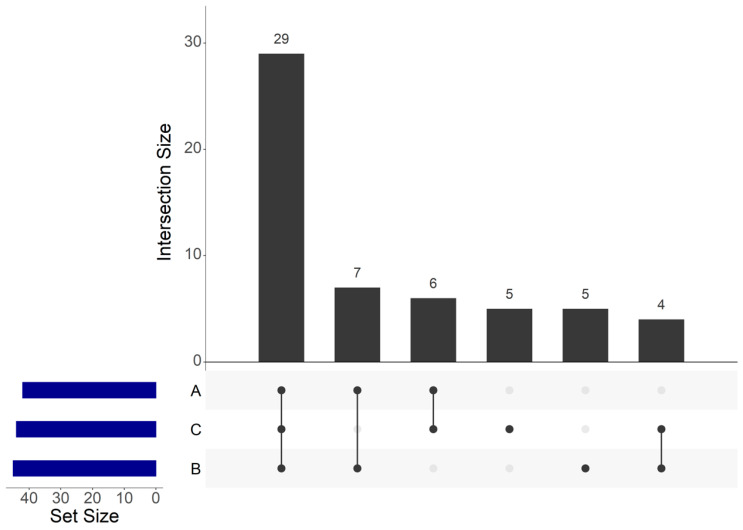
UpSet plot of shared operational taxonomic units (OTUs) identified within cold-smoked salmon vacuum-packed products according to the food processing factory.

Among the 56 OTUs, 29 core OTUs were identified in the three different factories (Figure 5), which were agglomerated in 12 genera composed by *Carnobacterium*, *Lactobacillus*, *Staphylococcus*, *Pantoea*, *Serratia*, *Proteus*, *Salinivibrio*, *Vibrio*, *Photobacterium*, *Shewanella*, *Psychrobacter* and *Pseudomonas*.

Seven core OTUs were identified between A and B samples. These seven OTUs were agglomerated in seven genera composed by *Brochothrix*, *Lactobacillus*, *Staphylococcus*, *Enhydrobacter*, *Psychrobacter*, *Marinimonas* and *Arcobacter*.

Six core OTUs between A and C samples were identified and agglomerated in six genera: *Carnobacterium*, *Lactobacillus*, *Staphylococcus*, *Serratia*, *Psychrobacter* and *Brevibacterium*. Four core OTUs between B and C samples were identified and agglomerated in four genera composed by *Carnobacterium*, *Aliivibrio*, *Photobacterium* and *Psychrobacter*. Five OTUs were only identified within B samples, which were agglomerated in three genera composed by *Carnobacterium*, *Serratia* and *Shewanella*. Five unique OTUs were also only identified within C samples, which were agglomerated in four genera composed by *Carnobacterium*, *Lactobacillus*, *Aerococcus* and *Pseudomonas*.

**Figure 6:**
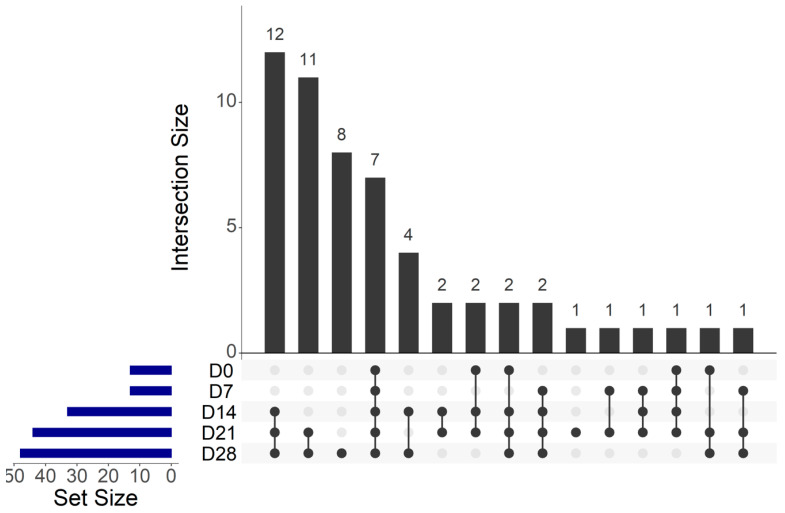
UpSet plot of shared operational taxonomic units (OTUs) identified within cold-smoked salmon vacuum-packed products during 28 days of storage.

Among the 56 OTUs, seven core OTUs were identified among the CSS products at each storage analysis date (Figure 6), which were agglomerated in six genera composed by *Staphylococcus*, *Vibrio*, *Photobacterium*, *Shewanella*, *Psychrobacter* and *Pseudomonas*. Twelve core OTUs were identified only at D14, D21 and D28, which were agglomerated in five genera composed by *Carnobacterium*, *Arcobacter*, *Enhydrobacter*, *Psychrobacter* and *Pseudomonas*. Eleven OTUs were unique to D21 and D28, which were agglomerated in seven genera composed by *Carnobacterium*, *Staphylococcus*, *Pantoea*, *Salinivibrio*, *Psychrobacter*, *Brevibacterium* and *Pseudomonas*. Finally, eight OTUs were unique to D28, which were agglomerated in six genera composed by *Carnobacterium*, *Lactobacillus*, *Aerococcus*, *Shewanella*, *Marinomonas* and *Pseudomonas*.

The genera *Brevibacterium*, *Marinomonas*, *Enhydrobacter* and *Arcobacter* belonged for their part to the subdominant population with a relative abundance below 0.05%.

Communities richness (observed OTUs) and evenness (Shannon diversity index) were assessed for all 84 samples and are summarized in Table 2. The storage time had an effect on both richness (*p* < 0.0001) and evenness (*p* < 0.0001). Communities were richer after 28 days of storage (with an average of 15.43 ± 4.95 OTUs). No richness differences were observed between D0 and D7 (respectively with an average of 4.56 ± 2.35 and 4.89 ± 1.83 OTUs). With regard to the evenness of communities, this was higher after 28 days of storage (with an average of 1.07 ± 0.54). Interestingly, the processing environment appeared to have no impact on either richness or evenness (respectively *p* = 0.60 and *p* = 0.83).

**Figure 7:**
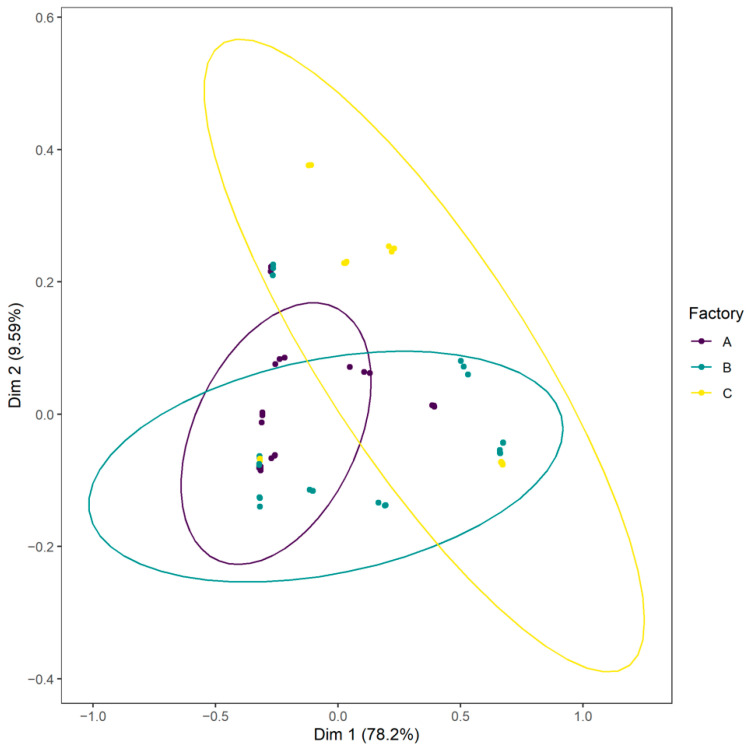
Weighted UniFrac principal coordinates analysis (PCoA) plot of CSS samples according to the food processing factory.

Weighted UniFrac principal coordinates analysis (PCoA) was generated to visualize samples (Figure 7). This PCoA highlighted shared taxa between samples, especially between factories A and B, but also differences according to the processing environment. PERMANOVA analysis based on weighted UniFrac distance showed that the processing environment, the storage date and the production batch influenced the bacterial community (respectively *p* < 0.0001) and explained respectively 17.6%, 14.2% and 45.7% of the sample microbiota differences.

## 4. Discussion

The first part of this study aimed to evaluate the culturable bacterial population of several CSS products processed in three different factories. The microbial load was high after one week of storage. The dominant population on D0 products consisted of Gram-negative *Vibrio*, *Enterobacteriaceae*, and Gram-positive LAB. The microbial load reached an average of 10^7^ CFU/g at the end of the experiment. These observations and the bacterial concentration were consistent with already published data. Indeed, Leroi et al. (1998) studied the microbial ecology of CSS during 35 days of storage at 8 °C [6]. The authors enumerated aerobic viable counts at a maximum of 10^6^ to 10^7^ CFU/g after 6 days of storage. In addition, Paludan-Müller et al. (1998) studied the role of LAB in vacuum-packed CSS spoilage [7]. The authors evaluated the total psychrotrophic viable counts during 7 weeks at 5 °C. Counts reached 10^6^ to 10^7^ CFU/g in two weeks and remained stable during the storage. Moreover, LAB growth did not seem to compete with Gram-negative bacteria as described by Leroi et al. (1998) [7].

Marine *Vibrio* such as *Photobacterium phosphoreum* were dominant among the bacterial populations of the different samples. This bacterium has already been described as a potential spoiler due to its ability to produce TMA from trimethylamine N-oxide (TMAO), which is known to be responsible for the typical strong fishy, urine and ammonia-like off-odors [3,41,42].

*Enterobacteriaceae* were dominant within B and C product communities. Psychrotrophic *Enterobacteriaceae* have been already identified on spoiled CSS, and particularly reported as dominant within injection brined products [5].

Lactic acid bacteria (LAB) such as *Carnobacterium maltaromaticum* and *Lactobacillus curvatus* have been widely described as dominant at a high level (10^7^–10^8^ CFU/g) on CSS products and could be involved in spoilage processes [43].

Interestingly, *Brochothrix thermosphacta* was enumerated on only one B product, yet on all C products. Several studies have reported the spoilage potential of this bacterium [5,44], notably able to produce butter/plastic/rancid, blue-cheese, sour/pungent off-odors, due to the high release of chemical compounds such as 2-heptanone and 2-hexanone [45,46]. More broadly, Stohr et al. (2001), by studying the inoculation of different spoilage bacteria on CSS (*Shewanella putrefaciens*, LAB, *Brochothrix thermosphacta*, *Aeromonas* spp., *Serratia liquefaciens*), were able to design a sensory and spoilage profile to better understand the CSS spoilage process and its major actors.

As described by Joffraud et al. (2006) in a study to evaluate CSS spoilage following different microbiota interaction, CSS spoilage due to metabolites production is often strain-dependent, which can explain the intraspecies diversity in terms of spoilage potential [9]. Furthermore, spoilage is also related to interactions, either between bacterial species, such as antagonistic or cooperative behavior, or between bacterial species and food matrices and the food processing environment. Indications of bacterial species interaction have been found in other food matrices, for example by Jaffrès et al. (2009), who studied the bacterial community in tropical cooked and peeled shrimps using a polyphasic approach (cultivable, non-cultivable and sensory analyses) [13]. These authors hypothesized that the spoilage process might be the result of interactions between *Brochothrix thermosphacta* and *Carnobacterium divergens*.

Chemical parameters (dry matter content, total fat, salt content and total phenols) were similar among the different samples and fluctuated during the experiment. These parameters were aligned with the NF V45-065 standard [47] on CSS properties.

Total fat significantly decreased after 28 days of storage. It is known that bacteria are able to degrade lipids. Notably, it has been reported that *Serratia*, *Staphylococcus* and *Pseudomonas* have the ability to degrade vegetable oil [48]. These genera are known to be part of the CSS microbiota. Their metabolic activities could explain this significant decrease of total fat.

Salt content was stable from 2.89 ± 0.41 g/100 g to 2.91 ± 0.67 g/100 g. It has been reported that despite its bacteriostatic effect, a low salt concentration could reduce the product sensory rejection limit and could not be sufficient to inhibit *Listeria monocytogenes* growth [5,49].

Total phenols were also stable during the storage period. In addition, no growth was observed at D28 on product A1. The total phenols on products from A at the beginning of the storage were higher than those on other products. The bacteriostatic effect of the smoking process may impact microbial growth or induce viable but non-culturable cells. Indeed, liquid smoke strongly affected growth and survival of *Listeria monocytogenes* [50]. Moreover, Neunlist et al. (2005), by assessing the impact of salting and cold-smoking processes on the cultivability of *Listeria monocytogenes*, showed a reduction of 2 Log CFU/g for inoculated processed salmon compared with raw salmon during 28 days of storage [51]. The authors also tested inoculation after the cold-smoked process and observed a 0.9 Log CFU/g reduction of the *Listeria monocytogenes* concentration on processed samples compared with unprocessed salmon within the first two weeks of storage. The concentrations of the control and processed samples were similar at the end of the storage period. Even if the authors did not highlight a viable but non-culturable state, the reduced concentration of *Listeria monocytogenes* in the processed samples during the first two weeks of storage, and the subsequent increase to reach the same concentration as the control, may indicate that the phenols compounds most probably stress bacterial cells but these cells later regain the ability to grow.

Total volatile basic nitrogen (TVBN) and trimethylamine (TMA) are considered as spoilage indicators. Their production increased significantly during the storage period. High TVBN concentrations suggest that CSS spoilage occurred after 14 days of storage. No strong differences in TVBN concentrations were observed across the different factories. However, as previously described in several studies, the use of TVBN alone as a relevant spoilage indicator must be put in perspective. In their study on CSS spoilage, Joffraud et al. (2006) found that *Vibrio* spp. produced a significant amount of TVBN although no off-odor was detected by a trained sensory panelist [9]. Furthermore, Brillet et al. (2005) showed that *Carnobacterium maltaromaticum* strains did not produce TVBN when inoculated in pure culture in sterile CSS, whereas when inoculated in naturally contaminated products, TVBN production was significantly enhanced [52]. Contrary to TVBN, TMA concentrations mostly increased on the products of A and B, suggesting that *Photobacterium phosphoreum* might be implicated in the spoilage process of these products [7,53].

Cold-smoked salmon can be re-contaminated during the manufacturing process through contact with contaminated surfaces (such as slicers, conveyors, etc.) [5]. The microbiota during storage may be different according to how and where products are processed. Metabarcoding analyses could help to explore this hypothesis.

Metabarcoding on 45 samples stored for 28 days and analyzed every seven days in triplicate allowed us to identify and to analyze the bacterial communities of nine CSS batches produced in three different factories. Out of over 129 sequenced samples, a total of 45 samples displayed low library sizes (<10,000 reads).

Bukin et al. (2019), by studying the effect of different 16S rRNA regions on bacterial communities monitored by metabarcoding, highlighted that the major bacterial diversity (covered by 95% of reads) could be achieved at a library size of 10,000 reads [54]. Thus, we decided to remove the 45 outliers for statistical purposes.

The dominant population identified confirmed already published data on CSS microbiota studied using cultivable methods, but also data from a few studies using culture-independent methods [5,7,18,22,55]. *Psychrobacter* is highly prevalent in our study (6.43 ± 18.20% of relative abundances). *Psychrobacter* occurrence seems to be widespread on seafood products and was also identified on raw salmon [19,44].

Thanks to the use of NGS, several studies on seafood have highlighted the high prevalence of *Psychrobacter* among seafood product spoilage bacterial communities [21]. Parlapani et al. (2018) used NGS to investigate the spoilage microbiota of thawed common cuttlefish (*Sepia officinalis*) stored at 2 °C [56]. The authors, by using an amplicon sequencing approach, highlighted that *Psychrobacter* was highly dominant among the samples, followed by *Pseudomonas*. In the literature, *Staphylococcus* is rarely described as a CSS dominant bacterium. Its occurrence is mainly due to exogenous origins such as salt, the aquatic environment or the processing environment [4,22]. As far as the *Enterobacteriaceae* family is concerned, the *Pantoea* genus is also rarely described in the CSS bacterial communities. *Pantoea* is an ubiquitous bacterium which has already been identified in aquatic environments [57]. Skrodenytė-Arbačiauskienė et al. (2008) analyzed the gut microbial diversity of 12 fish (six freshwater *Salmo salar* and six sea trout *Salmo trutta trutta*) using a cultural approach and 16S rRNA gene sequencing for colony identification [58]. The authors identified the genus *Pantoea* within the sea trout intestinal tract but not on salmon samples. In another study to assess bacterial resistance to the antibiotic oxytetracycline in Chilean salmon (*Salmo salar*) farming, Miranda and Zemelman (2002) identified a prevalence of *Pantoea* on fingerlings salmon samples [59]. In our study, the majority of this genus was found on A3 (19.4 ± 23.8%) and C1 (11.7 ± 12.8%). We assumed that the origin of *Pantoea* on these products could be explained by their aquatic farm origin or contamination during production. Twenty-nine OTUs agglomerated in 12 genera were identified as part of the core microbiota between all of the CSS products. All of these genera were part of the dominant population except for *Proteus*. González-Rodríguez et al. (2002) studied the microbial community of 54 batches of cold-smoked fish (30 CSS and 24 smoked trout) during three weeks of storage [60]. Colonies were counted and identified using API galleries. The authors identified *Proteus* as a dominant member of the *Enterobacteriaceae* family among the samples. Interestingly, in our study, *Aliivibrio* was not identified on A products, and *Brevibacterium* was not found on B samples. *Aerococcus* was only identified on C samples. In addition, *Arcobacter*, *Marinimonas*, *Enhydrobacter* and *Brochothrix* were not identified on C samples using metabarcoding. These results suggest the importance of the processing environment on the CSS microbiota, with a bacterial signature from this environment.

During the storage period, our findings highlighted that 12 OTUs (agglomerated in the following genera: *Carnobacterium*, *Arcobacter*, *Enhydrobacter*, *Psychrobacter* and *Pseudomonas*) were captured on D14; 11 OTUs (agglomerated in the following genera: *Carnobacterium*, *Staphylococcus*, *Pantoea*, *Salinivibrio*, *Psychrobacter*, *Brevibacterium* and *Pseudomonas*) were captured on D21; and eight OTUs (agglomerated in the following genera: *Carnobacterium*, *Lactobacillus*, *Aerococcus*, *Shewanella*, *Marinomonas* and *Pseudomonas*) were captured on D28. The emergence or capture of specific OTUs over time was also identified by Silbande et al. (2018) [61]. The authors studied the effect of different packaging atmospheres on the microbiological, chemical and sensory properties of tropical red drum (*Sciaenops ocellatus*) fillets stored for 29 days at 4 °C. The authors identified the emergence of *Leuconostoc* and *Lactococcus* after eight days of storage on fresh fillets that were vacuum-packed. These two OTUs were not identified at Day 0.

Alpha diversity analyses highlighted that the richness and evenness of the different CSS bacterial communities increased during the product storage period. However, no differences between the factories were observed. While we observed a global increase in the OTUs’ richness, Wiernasz et al. (2020) highlighted a reduction of the number of OTUs on salmon gravlax during 21 days of storage [55]. Salmon gravlax is a salt-sugar mixture with spices that is not treated using smoke or heat. This particular treatment may lead to competitive flora which become dominant on these products which are not found on the standard cold-smoked process.

Beta diversity analyses and weighted UniFrac PCoA confirmed a core microbiota but also highlighted differences in communities, specifically between A products and C products. In addition, we identified five OTUs (agglomerated in *Carnobacterium*, *Serratia* and *Shewanella*) specific to B and five others specific to C (agglomerated in *Carnobacterium*, *Lactobacillus*, *Aerococcus* and *Pseudomonas*). These results strengthened the specific factory signature observation. Our findings showed that the different compositions of CSS microbiota were affected by the processing environment and the length of storage but also the production batch. This clearly confirms that even if a core community existed between the samples, the processing factory had a bacterial signature composed by spoilage organisms which can contaminate CSS products during processing, attesting to the importance of the processing environment for the quality and shelf-life of CSS.

Rouger et al. (2018) observed identical results in their study of chicken leg microbiota, where two chicken leg samples from two different batches, stored under modified atmosphere packaging, showed similar microbiota [62]. Interestingly, these two samples were processed in the same slaughterhouse on the same day. These results strengthen the hypothesis of a food processing bacterial signature on the microbial communities of products. To investigate this environmental influence, Stellato et al. (2016) compared fresh meat microbiota with environmental samples from small and large-scale retail butcheries [63]. The authors highlighted 48 core genera shared between product and environment samples. Among these 48 genera, *Pseudomonas* spp., *Brochothrix* spp., *Psychrobacter* spp., *Streptococcus* spp. and *Acinetobacter* spp. were identified. These genera were reported as members of the meat spoilage community, highlighting the importance of the surface microbiota on product quality. By using a polyphasic approach (cultivable method with bacterial identification using the 16S rRNA gene and non-cultivable methods using NGS), Møretrø et al. (2016) identified the processing environment as a source of spoilage genera *Pseudomonas* and *Shewanella* [19]. Phylogenetic analyses based on part of the 16S rRNA gene demonstrated the transfer of *Pseudomonas* from processing samples to salmon fillets, thus strengthening the links between the processing environment and product samples and the impact of the processing environment on the shelf-life.

## 5. Conclusions

In this study, we described the microbiota of vacuum-packed cold-smoked salmon products produced in three different factories and stored for 28 days. We used a polyphasic approach composed of cultivable methods and non-cultivable methods. The use of metabarcoding did not highlight unexpected genera except for *Pantoea*, and our findings were consistent with already published cultivable data on CSS bacterial communities. However, a next-generation sequencing-based approach highlighted the emergence of operational taxonomic units during product storage and provided insights on the CSS microbial ecology. A core microbiota composed of spoilage bacteria was shared by the 45 products but strong differences linked to the processing environment were observed. Indeed, we found that CSS products bore a factory bacterial signature. These results were obtained from three different processing plants and 45 samples and must be considered at this scale. This suggests the importance of the processing environment on food safety and quality. A better understanding and characterization of surfaces and residential bacteria and their dynamics using metabarcoding approaches may be a key to gaining greater insight into a factory’s “health condition” to improve food safety and quality management.

## Figures and Tables

**Table 1:** Cold-smoked salmon samples description (processing factory, production batch, origin and use-by date).

Factory	Production	Origin/Label	Use-by-Date
A	A1	Scotland	13 March 2019
	A2	Norway	13 March 2019
	A3	Norway	07 March 2019
B	B1	Scotland	09 March 2019
	B2	Norway	09 March 2019
	B3	Scotland/Label Rouge	04 March 2019
C	C1	Scotland	15 March 2019
	C2	Norway	15 March 2019
	C3	Ireland/Organic	09 March 2019

**Table 2:** Observed richness and evenness for 16S rRNA amplicons analyzed in this study. Data are expressed in Mean ± SD.

Factory	Salmon	Date	Observed OTUs	Shannon Index
A	A1	D0	4.000 ± 0.000	0.006 ± 0.001
A	A1	D14	1.333 ± 0.577	0.000 ± 0.001
A	A1	D21	2.667 ± 1.155	0.004 ± 0.002
A	A2	D14	12.667 ± 0.577	1.130 ± 0.018
A	A2	D21	12.000 ± 1.000	0.576 ± 0.020
A	A2	D28	18.667 ± 1.528	0.501 ± 0.016
A	A3	D7	4.000 ± 1.000	0.221 ± 0.023
A	A3	D14	8.000 ± 1.000	0.400 ± 0.065
A	A3	D21	9.000 ± 0.000	0.902 ± 0.010
A	A3	D28	16.333 ± 1.155	1.333 ± 0.018
B	B1	D7	3.667 ± 1.155	0.008 ± 0.001
B	B1	D21	11.000 ± 1.000	0.807 ± 0.006
B	B1	D28	5.667 ± 1.155	0.518 ± 0.012
B	B2	D0	7.333 ± 1.528	0.664 ± 0.006
B	B2	D7	7.000 ± 1.000	0.135 ± 0.013
B	B2	D14	7.333 ± 0.577	0.428 ± 0.026
B	B2	D21	13.000 ± 1.732	0.537 ± 0.040
B	B3	D0	2.333 ± 0.577	0.002 ± 0.002
B	B3	D14	12.000 ± 0.000	0.678 ± 0.054
B	B3	D21	14.000 ± 1.000	1.074 ± 0.022
B	B3	D28	17.333 ± 0.577	1.080 ± 0.027
C	C1	D14	10.333 ± 0.577	0.060 ± 0.002
C	C1	D21	7.000 ± 1.000	0.054 ± 0.005
C	C1	D28	11.667 ± 1.155	0.539 ± 0.002
C	C2	D21	13.667 ± 1.528	0.337 ± 0.062
C	C2	D28	17.667 ± 1.528	1.744 ± 0.007
C	C3	D21	8.333 ± 2.309	0.816 ± 0.014
C	C3	D28	20.667 ± 0.577	1.800 ± 0.004

OTUs: operational taxonomic Units.

## Data Availability

The data presented in this study and statistical outputs are openly available in Mendeley Data at DOI:10.17632/wz6jkfdy48.1 and in Data INRAE at DOI:10.15454/BIVUOQ.

## Supplementary Materials

The following are available online at https://www.mdpi.com/2304-8158/10/2/362/s1, Table S1: Bacterial growth evolution of lactic acid bacteria (LAB), *Enterobacteriaceae*, *Brochothrix thermosphacta*, *Vibrio* and total viable psychrothropic count (TPVC) in vacuum-packed cold-smoked salmon (CSS) products during 28 days of storage.
